# Supplementary figures and images for: A kinesin-13 family kinesin in Trypanosoma brucei regulates cytokinesis and cytoskeleton morphogenesis by promoting microtubule bundling
Source: PLoS Pathog. 2024 Feb 1;20(2):e1012000. doi: 10.1371/journal.ppat.1012000 (PMC10863849; doi:10.1371/journal.ppat.1012000)

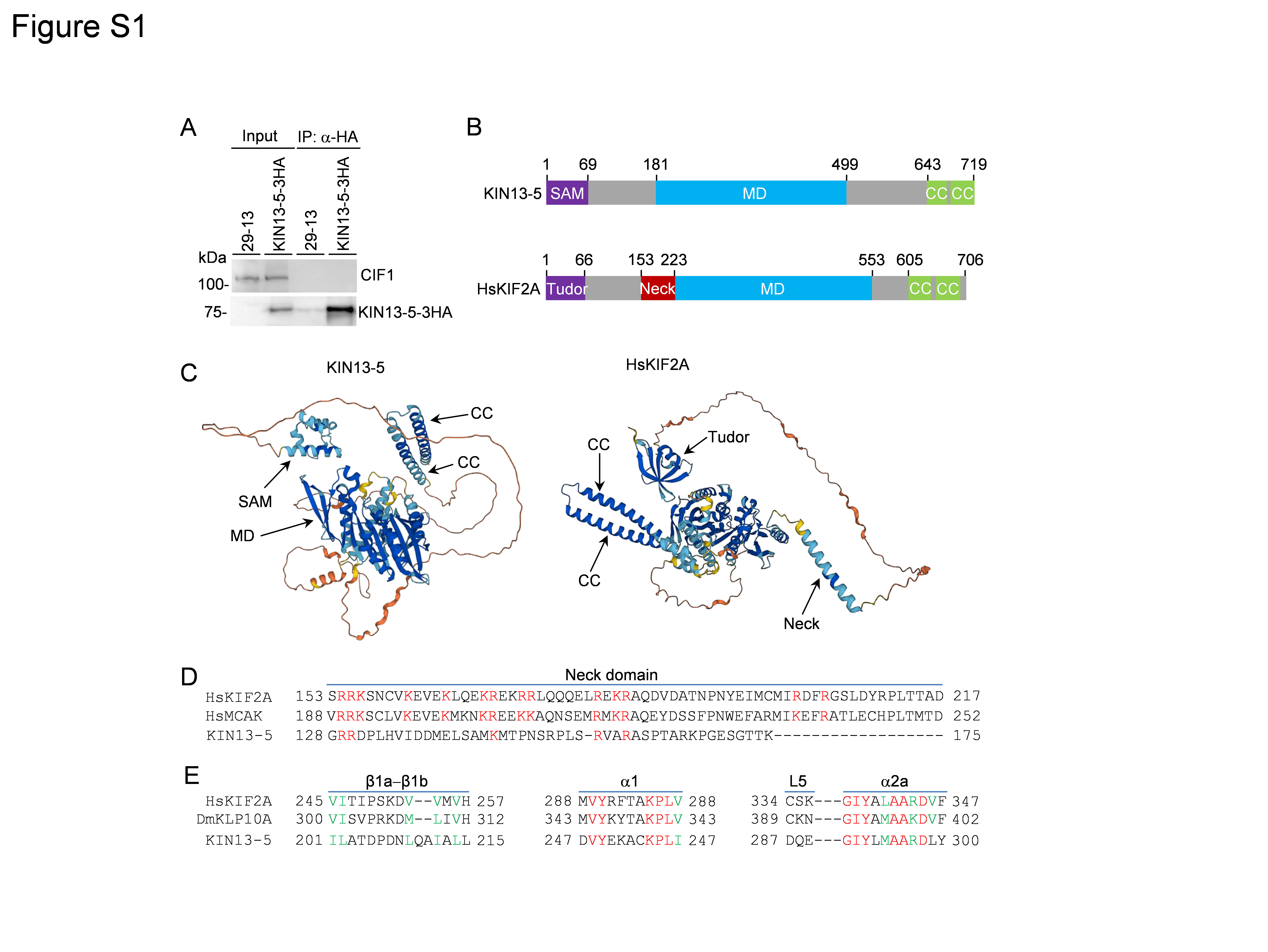

Supplement: S1 Fig — (A). Co-immunoprecipitation to test the interaction between KIN13-5-3HA and CIF1. Immunoprecipitation (IP) was performed by incubating cell lysate with anti-HA affinity gel, and western blotting was performed to detect CIF1 and KIN13-5-3HA with anti-CIF1 antibody and anti-HA antibody, respectively. (B). Schematic drawing of the structural domains in T. brucei KIN13-5 and human KIF2A. SAM, sterile alpha motif; MD, motor domain; CC, coiled coil. (C). Predicted structure of KIN13 and HsKIF2A by AlphaFold. (D). Alignment of the neck domain from HsKIF2A, HsMCAK, and T. brucei KIN13-5. The residues highlighted in red indicate the conserved positively charged residues that are required for stimulating kinesin microtubule-depolymerizing activity. (E). Alignment of the second microtubule-binding motif within the motor domain from HsKIF2A, DmKLP10A, and T. brucei KIN13-5. Identical and conserved residues are highlighted in red and green, respectively. (TIF) [file ppat.1012000.s001.tif]
