# Supplementary material for: A kinesin-13 family kinesin in Trypanosoma brucei regulates cytokinesis and cytoskeleton morphogenesis by promoting microtubule bundling
Source: PLoS Pathog. 2024 Feb 1;20(2):e1012000. doi: 10.1371/journal.ppat.1012000 (PMC10863849; doi:10.1371/journal.ppat.1012000)
Supplement: Attachment — Submitted filename: Response to reviewers.docx [file ppat.1012000.s003.docx]

Response to reviewers’ comments

**Manuscript Number**: PPATHOGENS-D-23-01674
**Title**: A kinesin-13 family kinesin in Trypanosoma brucei regulates cytokinesis and cytoskeleton morphogenesis by promoting microtubule bundling

**Authors**: Huiqing Hu, Yasuhiro Kurasawa, Qing Zhou, and Ziyin Li

We very much appreciate the most insightful comments and the very useful suggestions made by the two experts. We have revised the manuscript accordingly. The following is a point-by-point response detailing how we have dealt with the points raised by the reviewers.

**Part I - Summary**
Please use this section to discuss strengths/weaknesses of study, novelty/significance, general execution and scholarship.

**Reviewer #1:**

In this manuscript, the authors have discovered a new molecular component of the machinery controlling cytokinesis in the protist Trypanosoma brucei. It is a kinesin of the family 13 and it turns out to contribute to positioning of the cleavage furrow. Surprisingly, it does not seem to crop microtubules as most members of this family but rather, it bundles them, what could be critical during cytoskeletal remodelling at cytokinesis. As a reminder, trypanosomes do not depolymerise their cytoskeleton during cell division, so it makes a lot of sense.

The authors use well-established tools during the investigation: BioID to identify new components of the cytokinesis pathway, localisation using tagged fusion proteins, functional genomics by RNAi and electron microscopy including whole-mount cytoskeletons. In addition to that, they produce in vitro data showing the bundling activity of the new kinesin.

This combination results in a solid and well-controlled study, with nice and clear figures and a new message, further refining our molecular knowledge of cell division in trypanosomes. It will be of interest for parasitologists and also cell biologists interested in cytokinesis.

Thanks very much for taking your busy time to review our manuscript and for providing constructive comments.

**Reviewer #2:**

The authors identify a largely uncharacterized Kinesin-13 family protein (KIN13-5) as an interactor of components of the trypanosome flagellar attachment zone. They localise the protein to both posterior and anterior ends of the cell and show that depletion of the protein results in defects in cytokinesis with loss of organisation of the microtubules at the posterior and to a lesser extent anterior end. They also present data from recombinant protein assays suggesting that the protein is multimeric and able to bundle microtubules. Finally, they show it’s localisation to the anterior site is dependent on FPRC.

The work adds to the list of components of the cleavage furrow in trypanosomes (including a non-processive dimeric kinesin KLIF with strong evidence for MT bundling [PMID: 36787745]), but the bigger impact would be in understanding whether this protein was acting primarily at anterior or posterior end (and if the latter, how?) and also in elucidation of the evolution of an MT-bundling kinesin from the depolymerizing Kinesin-13 family. There’s lots of work on Kinesin-13 family members, especially MCAK and KIF2A, and where known they are dimeric depolymerizing motors, so the finding of a Kinesin-13 that is non-depolymerizing but which bundles MTs would be very interesting – especially if some insight could be found on the features of the protein necessary (binding site, whether ATPase dependent, how linking MTs, etc.). However, the work doesn’t bring much data on the mode of action of KIN13-5 in cytokinesis, focusing mainly on the interaction at the furrow ingress site (anterior end) when the major defect appears to be at the posterior end. Moreover, the data on bundling do not provide sufficient evidence to support the authors’ conclusions and should in my opinion be removed or subject to major revision. Without either more information on mode-of-action or conclusive data on bundling activity I afraid I don’t think the work brings sufficient new insight to cytokinesis in trypanosomes.

Thanks very much for providing constructive comments to help us revise the manuscript. Please see below the response from us to your comments.

**Part II – Major Issues: Key Experiments Required for Acceptance**
Please use this section to detail the key new experiments or modifications of existing experiments that should be absolutely required to validate study conclusions.

**Reviewer #1:**

I have one question and one comment.

Question. Can the authors exclude the fact that KIN13-5 could have depolymerising activity? Based on the images shown at figure 5, it seems that the posterior end of knockdown cells contains almost twice as many microtubule ends (about 22, 5E) compared to the control (about 13, 5A). It is even more pronounced at Fig. 5G. I appreciate that KIN13-5 bundles microtubules in vitro (Fig. 6) but the source of these is not described in M&M. Are they from trypanosomes or from another organism? The cellular context (with MAPs for example) might be different from the in vitro situation. I don’t know how representative are these images but based on them, it remains a possibility that KIN13-5 could also restrict microtubule elongation and/or reduce microtubule length, hence facilitation the formation/progression of the cleavage furrow. I would be happy to hear the authors’ opinion on this point.

This is a great question. It is true that the posterior end of the KIN-13-5 knockdown cells possesses more microtubules (Fig. 5) than that of the non-induced control cells. Is it possible that KIN13-5 still possesses microtubule depolymerizing activity? Based on the *in vitro* assays, which we have performed numerous times (we started the experiments two years ago, and we repeated >30 times), we do not observe depolymerizing activity for KIN13-5 (please see the new data in Fig. 7). However, we are always able to detect depolymerizing activity for KIN13-1. Although we cannot confidently exclude the possibility that KIN13-5 possesses depolymerizing activity, at least from our *in vitro* assays we are unable to detect depolymerizing activity for KIN13-5. As for why there are more microtubules at the posterior end, we think this is due to unequal division of the cytoskeleton of the mother cells, resulting in the generation of a new-flagellum daughter cell with less microtubules and an old-flagellum daughter cell with more microtubules. The KIN13-5 RNAi cell shown in Fig 5E is very likely to be the old-flagellum daughter cell after unequal cell division. For the cell in Fig. 5G, the posterior end has more microtubules, because the nascent posterior end of the old-flagellum daughter cell is positioned next to the existing posterior end of the new-flagellum daughter cell. So, the posterior end of this cell contains the microtubules from both the existing posterior and the nascent posterior. Please see Fig. 3 for the position of the nascent posterior in KIN13-5 RNAi cells.

Because the posterior of KIN13-5 RNAi cells do not have strong YL 1/2 immunostaining, it is unlikely that KIN13-5 could restrict microtubule elongation and/or reduce microtubule length.

The source of the tubulins used for microtubule assembly and assays has been provided in the M&M section.

Comment. Throughout the manuscript, it is written that the kinesin KD divide ‘asymmetrically’ while control cells would divide ‘symmetrically’ and (see for example fig. 7). This is not completely correct as in normal procyclic cells, the new flagellum is shorter than the old one at cytokinesis and that the daughter cells have different morphologies. This is actually visible on several images in the paper (Fig. 3-5) and has been well reported in the literature (see for example Abeywickrema et al. Mol Mic2019). This is confusing, I understand the authors want to highlight the difference but I would strongly advise to use a different wording to describe the control situation.

This is another great point that is very well taken. We are fully aware of the “asymmetrical” division of the wild-type trypanosome procyclic cells. We now used “normal” cytokinesis for the control cells and “abnormal” cytokinesis for KIN13-5 RNAi cells (Fig. 2H). In the text, we used “significantly unequal” cytokinesis to describe the cytokinesis in KIN13-5 RNAi cells. We cited the Abeywickrema paper (Mol Micro, 2019) to explain that the control trypanosomes actually do not actually divide in a totally equal manner, but the KIN13-5 RNAi cells divide in such an unequal manner that the cell body size of the new-flagellum daughter cell is much smaller (lines 21-25, page 7). We hope these information and explanation will help the readers understand the difference between control and KIN13-5 RNAi cells.

**Reviewer #2:**

1) MT bundling activity.

There is a fundamental and important distinction between a protein that bundles MTs and a preparation that aggregates them. Lots of poorly folded or otherwise misprocessed proteins expressed in bacteria will cause MTs to aggregate, but this has no relevance to the biological roles of the properly processed proteins in cells. For this reason it is usual for material going into MT assays to be highly clarified (>200,000g) to remove aggregates and also for there to be supporting evidence to suggest the remaining protein is correctly folded, active, and/or in some other way relevant to material from the organism in question. Neither seems to be the case here. In fact, in this case the native gels strongly suggest the presence of large amounts of material of heterogeneous size/shape/stoichiometry which would be indicative of misfolded protein (Fig. 6D). The amount of these seems to correlate well with the degree of MT clustering in the microscopy data (Fig. 6E) and there’s no evidence given for MT-stimulated ATPase activity that would be at least be indicative of folding of some of the motor domains in the preparation.

In addition, the turbidity assays do not seem to be interpretable (Fig. 6F). The level of noise in each sample is substantially higher than I have seen in comparable assays looking at bundling/depolymerization (e.g. PMID: 17951709, 21723831, 31280993). The baseline for each sample jumps weirdly on addition of what should be identical preparations of MTs at time zero. The depolymerizing KIN13-1 does not enhance loss of turbidity over MTs alone (which would be expected given depolymerizing activity), whereas the ‘bundling’ MT KIN13-5 does not increase turbidity at all over time – but KIN13-5 alone does. None of these data seem to make biological sense and the data seem to bear little relationship to the observations in the MT microscopy.

In all, I don’t think these data can be considered sufficient support to make the important novel claim that KIN13-5 bundles MTs. The flow chamber imaging might be more convincing if were ATP-dependent (although in this case the suggestion is that it is motor-independent), or if it was titratable with highly clarified material, or if specific point mutants remove the activity with the same level of apparent misfolding/aggregation, etc. etc.

These are great points that are very well taken. We have performed the experiments following the suggestions by clarifying the purified proteins using ultracentrifuge at >200,000g. We also re-prepared the microtubules according to published protocols using ultracentrifugation to prepare microtubule seeds and then assemble longer microtubules from the seeds, etc. It is true that in our previous experiments there are some aggregates for some of the purified proteins. Now with the highly clarified proteins, we observed a homogenous size of the proteins on the blue native gel. Using these highly clarified proteins, we were able to reproduce the microtubule bunding assays. We set up different concentrations of the purified proteins, and also included several control proteins. For the turbidity assays, there were some technical issues with the turbidity assays previously, because of the small volume of samples used in the cuvette. We have decided to remove this data.

We hope that this new experimental data provided convincing evidence to prove that KIN13-5 has microtubule bundling activity in vitro.

2) Mode of action of KIN13-5 in cells.

Knockdown of KIN13-5 produced a defect in ‘placement’ of the cytokinetic furrow, but without more data on the in vitro activity of KIN13-5, I felt there was insufficient insight added by these data. KIN13-5 localises to both anterior and posterior sites. The authors show the interaction at the anterior site might be FAZ dependent, but as far as I could tell there was no defect in cytokinesis initiation (at the anterior). Instead, the major defect seems to be formation of the posterior end. As such, the key questions seem to be the role/activity of KIN13-5 at the anterior versus posterior sites and how this translates to furrow placement. How does lack of KIN13-5 cause furrow misplacement? Is there any change in furrow initiation as a result of lack of KIN13-5 at this position? What is KIN13-5 binding at the posterior sites? Is the activity and/or localisation dependent on motor activity? Does KIN13-5 link MTs in the cell? Does it bind to the +ve of MTs as other Kinesin-13s?

I’m certainly not suggesting all of these questions need to be addressed in this manuscript, but I felt that in the absence of robust data on in vitro activity, some of these issues would need to be address to warrant publication in PLoS Path.

These are all great points! Thanks for bringing these up to enlighten us for in-depth characterization of KIN13-5. The phenotype caused by KIN13-5 RNAi is correlated with the loss of microtubule bundling activity of KIN13-5. The lack of microtubule bunding at the posterior cell end of KIN13-5 RNAi cells suggests the loss of KIN13-5 microtubule bundling activity after RNAi. The localization of KIN13-5 to the posterior end and the *in vitro* microtubule bunding activity for KIN13-5 support the function of KIN13-5 at the posterior end. The defect in microtubule bundling at the anterior end of the new-flagellum daughter cell is often difficult to record by EM, because 1) the microtubules at the anterior end are often hidden behind the old-flagellum daughter cells, and 2) there are not many cells that are just prior to cytokinesis initiation in KIN13-5 RNAi population (cytokinesis initiation still occurs and there are many non-equally dividing cells). However, we were able to detect some cells before cytokinesis initiation and at very early stages of cytokinesis, and we were able to detect loss of microtubule bundling (Fig. 5F, G). Additionally, the function of KIN13-5 at the anterior end of the new-flagellum daughter cell can be deduced from the RNAi results (Figs. 2 and 3), which showed that KIN13-5 RNAi cells divide in a significantly unequal manner, and the new-flagellum daughter cell has varying sizes, indicative of mis-placed cleavage furrow. The smaller size of the new-flagellum daughter cell indicates that it has less microtubules. If we assume that in order for wild-type cells to divide equally, the furrow has to initiate from the anterior end of the new-flagellum daughter cell by bundling a certain number (or fixed number, let’s say 30 microtubules) of microtubules into the new-flagellum daughter cell. In KIN13-5 RNAi cells, due to the loss of KIN13-5 activity, microtubules are not bundled at the anterior end, so the furrow can initiate randomly by separating varying numbers of microtubules (let’s say 5, 10, 12, 16, or 20 microtubules) into the new-flagellum daughter cell. This can explain why the new-flagellum daughter cells of dividing KIN13-5 RNAi cells have varying sizes and are sometimes very skinny (Fig. 2E). Based on these results, we interpreted that KIN13-5 is required for correct cleavage furrow placement. KIN13-5 is not required for cytokinesis initiation or furrow ingression. We predict that KIN13-5 also binds to the plus-tip of microtubules, based on the observation that KIN13-5 localizes to the posterior cell end where plus ends of the subpellicular microtubules are located. At the anterior cell tip, KIN13-5 might binds MtQ, whose plus ends are directed at the anterior cell end. However, we have not performed experiments to test this.

For the other questions raised by this reviewer, they are all great questions, and we are planning to further characterize KIN13-5 by biophysical approaches in the future. For the question of how KIN13-5 bundles microtubules, it is possible that it may do so through the mechanism similar to the *Drosophila* kinesin-13 family protein KLP10A, which forms spirals and ring complexes around the MTs (Tan et al., Structure, 2008; Zhang et al., PLoS One, 2013), although the precise mechanism for KIN13-5 remains to be experimentally explored.

We believe that the most critical experiment in this work is the microtubule bundling assay, which this reviewer has made excellent suggestions for us to repeat the experiment using highly clarified recombinant proteins. We additionally re-prepared microtubules by following the well-established protocols from the Vale lab (UCSF), resulting in better quality of the assembled microtubules. Importantly, we were able to reproduce the microtubule bundling activity for KIN13-5 and the microtubule depolymerizing activity for KIN13-1. We also included TbSpef1 as a control, and we were able to detect its microtubule bundling activity, as reported previously by Dr. Cynthia He’s lab. Overall, we believe that this work adds new insights into the regulation of cytokinesis in trypanosomes. As for why a kinesin-13 family protein has the microtubule bundling activity, we think that this comment should be carefully evaluated. First, how reliable one can group KIN13-5 into the kinesin-13 family? We are not bioinformaticians, and do not have expertise to comment on the previous bioinformatics analysis. On the other hand, do all kinesin-13 family kinesins necessarily have to possess microtubule-depolymerizing activity? Are there exceptions? There are reports that the *Drosophila* kinesin-13 family protein KLP10A can bundle microtubules.

**Part III – Minor Issues: Editorial and Data Presentation Modifications**
Please use this section for editorial suggestions as well as relatively minor modifications of existing data that would enhance clarity.

**Reviewer #1:**

Please provide the source of rhodamine-labelled tubulin (which organism, how purified) (page 11 line 16 and page 21).

This information has been added to the revised manuscript in the Material and Methods section.

**Reviewer #2:**

It is absolutely essential to interpretation of the data in Fig 6 to know the ratio of tubulin to recombinant protein. I could find no information on the amount of recombinant preparation used in the turbidity assays. The work also talks about concentrations of microtubules, but no information is given on distribution of MT lengths as would be required to calculate this, and the amount stated equates to ~640mg/ml protein for 1µm MTs. I assume concentration of tubulin is meant not MTs?

We have now included the concentration of the recombinant proteins in the microtubule bundling assay experiments, as well as the concentration of tubulins in the microtubules. This reviewer is correct that the concentration of MTs provided in the manuscript is actually the concentration of tubulins, which was calculated by running the proteins of the assembled microtubules on SDS-PAGE.

Pseudocoloring of the MT microscopy (Fig.6E) to red on black when there is only one channel presented only serves to make the MTs very difficult to see. Please change to grayscale (ideally inverted).

We have changed them to grayscale. Thanks.
